# Natural products exert anti-tumor effects by regulating exosomal ncRNA

**DOI:** 10.3389/fonc.2022.1006114

**Published:** 2022-09-20

**Authors:** Shuang Hu, Yi Liu, Shuguang Guan, Zhidong Qiu, Da Liu

**Affiliations:** School of Pharmacy, Changchun University of Chinese Medicine, Changchun, China

**Keywords:** natural products, exosomes ncRNAs, cancer treatment, biomarkers, exosomes vector

## Abstract

Currently, more than 60% of the approved anti-cancer drugs come from or are related to natural products. Natural products and exosomal non-coding RNAs (ncRNAs) exert anti-cancer effects through various regulatory mechanisms, which are of great research significance. Exosomes are a form of intercellular communication and contain ncRNAs that can act as intercellular signaling molecules involved in the metabolism of tumor cells. This review exemplifies some examples of natural products whose active ingredients can play a role in cancer prevention and treatment by regulating exosomal ncRNAs, with the aim of illustrating the mechanism of action of exosomal ncRNAs in cancer prevention and treatment. Meanwhile, the application of exosomes as natural drug delivery systems and predictive disease biomarkers in cancer prevention and treatment is introduced, providing research ideas for the development of novel anti-tumor drugs.

## Introduction

Cancer has become a major disease that threatens human health and safety. The American Cancer Society (ACS) released the Cancer Statistics 2022 report stating that prostate, breast, lung, and colorectal cancers are the most prevalent cancers. However, the number of cancer deaths in the U.S. dropped 32% compared to 1991 (1), and the use of innovative drugs has directly impacted the survival rate of cancer patients (1–3).

Natural products include components and metabolites of plants, animals, insects, marine organisms and microorganisms (4), and many endogenous chemicals in humans and animals can also be used as natural products (5, 6). Many of them have been shown to have the ability of anti-inflammatory, anti-cancer, antioxidant, antibacterial and antiviral (7). Moreover, there exist studies showing that the natural products can promote the apoptosis, inhibit the migration and proliferation to exert anti-cancer effects (4). And the natural products have the advantages of easy to apply, low cost, easy to obtain and acceptable therapeutic method with minimal cytotoxicity (8). Therefore, it is of great significance to study the therapeutic effect of natural products on cancer.

Exosomes are endogenous components of organisms released by a variety of cells that contain ncRNAs that are genetically linked and involved in cancer development and progression (9, 10). Moreover, it can serve as a delivery system to deliver a variety of stuff such as drugs to cells (11). Nowadays, there are a growing number of researches detailing the mechanism of action of natural products through the regulation of exosomal ncRNAs to produce anticancer efficacy (12). In this review, the pharmacological effects of natural products and the anti-tumor mechanism of exosomal ncRNAs induced by natural products are clarified.

## Pharmacological effects of natural products against cancer

Research on natural products began in the 1990s. In recent years, with the development of scientific research technology, countries are paying more and more attention to the research and development of new natural products, and the research and development of natural products has shown a rapid growth trend (13). According to relevant information, natural products research hotspots focus on plants, animals and microorganisms on land and in the sea, involving a variety of components including proteins, peptides, amino acids, alkaloids and antibiotics (14, 15).

Natural products have a wide range of promising applications in the field of antitumor research. Traditional antitumor drugs act by affecting DNA synthesis and cell mitosis. Therefore, compared with natural antitumor drugs, traditional antitumor drugs have higher toxicity and side effects and are less selective (16). The development of natural antitumor drugs has not only ensured the efficacy of resulting in many innovative drugs with higher targeting for clinical practice (17).

For cancer treatment, drugs that directly inhibit the growth of tumor cells or promote their apoptosis are the most direct ways to fight cancer. Natural products can kill tumor cells through targeted aggregation, and also take advantage of qualitative differences between tumor cells and normal cells and molecular biological differences to target tumors, thereby inhibiting tumor cell proliferation (18). The biological processes involved in this process include signal transduction, cell fusion, and multiple types of metabolic pathways. In addition, natural products can reduce platelet aggregation, prevent cancer cell retention and inhibit cancer cell metastasis (19). It has been reported that the natural products may improve the local hypoxia of solid tumors by affecting microcirculation and increasing vascular permeability, which improves the sensitivity of treatment (20). Besides, natural products can produce some series of biological effects to exert anti-cancer pharmacological effects, such as promoting active ingredients, immune cells and cytotoxins to reach the tumor site (21). Studies have shown that natural products improve cellular immune function by increasing the body’s complement level, and reduce treatment-induced tissue fibrosis by inhibiting the formation of rough fibroblasts. Natural products can regulate the immune status of the body by promoting the phagocytosis of the reticuloendothelial system and enhance the resistance of the body to external malignant stimuli (22, 23).

Moreover, the natural products can also help to improve the drug resistance of tumors. Methods to reduce drug resistance of tumor cells are also the current focus of researchers (24). Cellular drug resistance is caused by the increase of target enzymes or the change of the affinity of target enzymes to anticancer drugs during the treatment of anticancer drugs, resulting in weakened drug activity, accelerated drug inactivation and rapid DNA repair, which affect the absorption or excretion of anticancer drugs (25). Research approaches reported in recent years to address drug resistance in tumor cells include combining anti-cancer drugs, eliminating the reversibility of anti-cancer drugs for cancer treatment, and affecting the tumor microenvironment (TME) (26–28). In addition, drug combination application and personalized medication guidance are also significant to eliminate tumor drug resistance (29, 30). Natural products may also enhance the effects of radiotherapy through mechanisms such as enhancing hormonal regulation, promoting pituitary-adrenocortical function, and increasing the relative value of cyclic adenosine monophosphate (31).

## Exosomes ncRNAs regulate cancer progression

**Figure 1:**
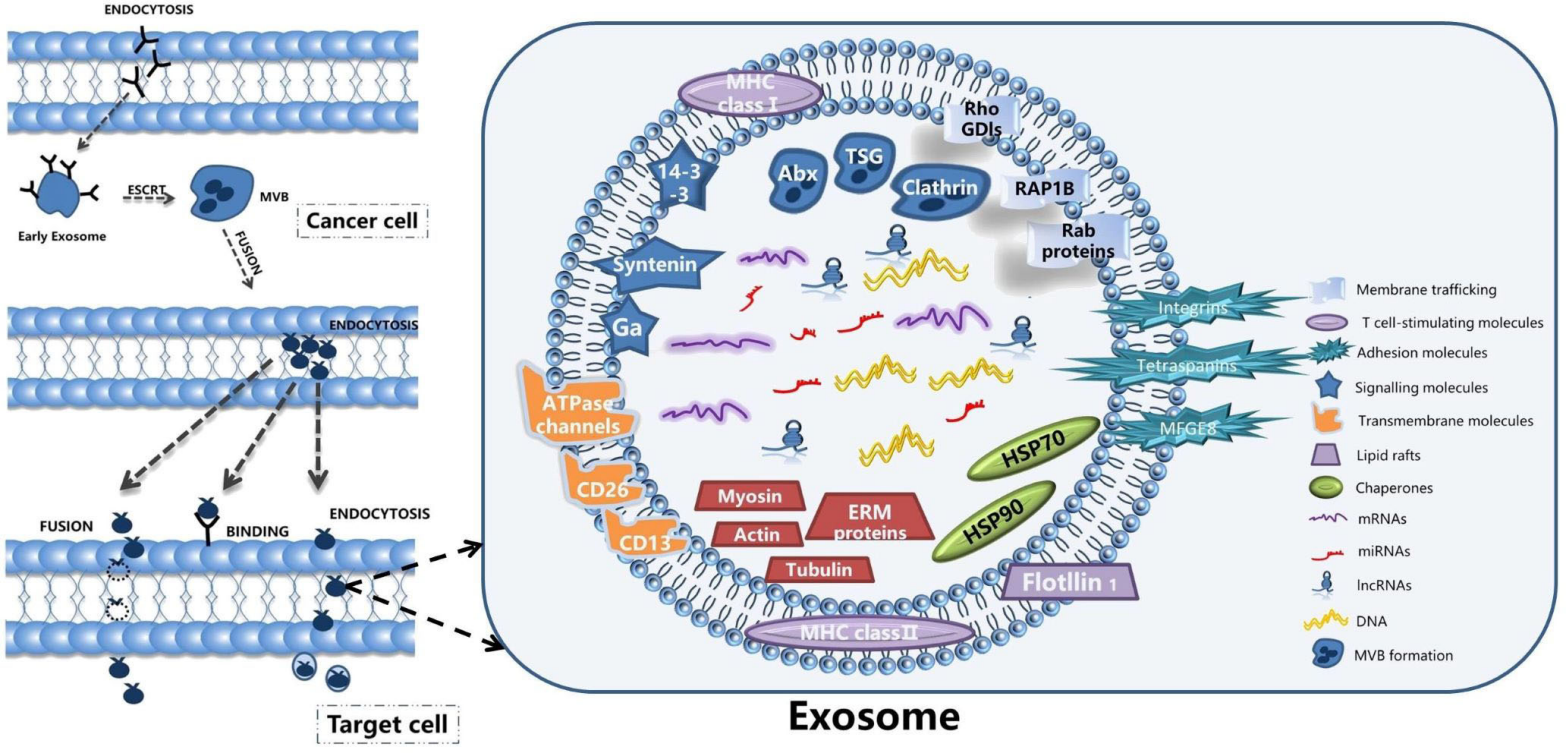
The structure and secretion mode of exosomes.

**Figure 2:**
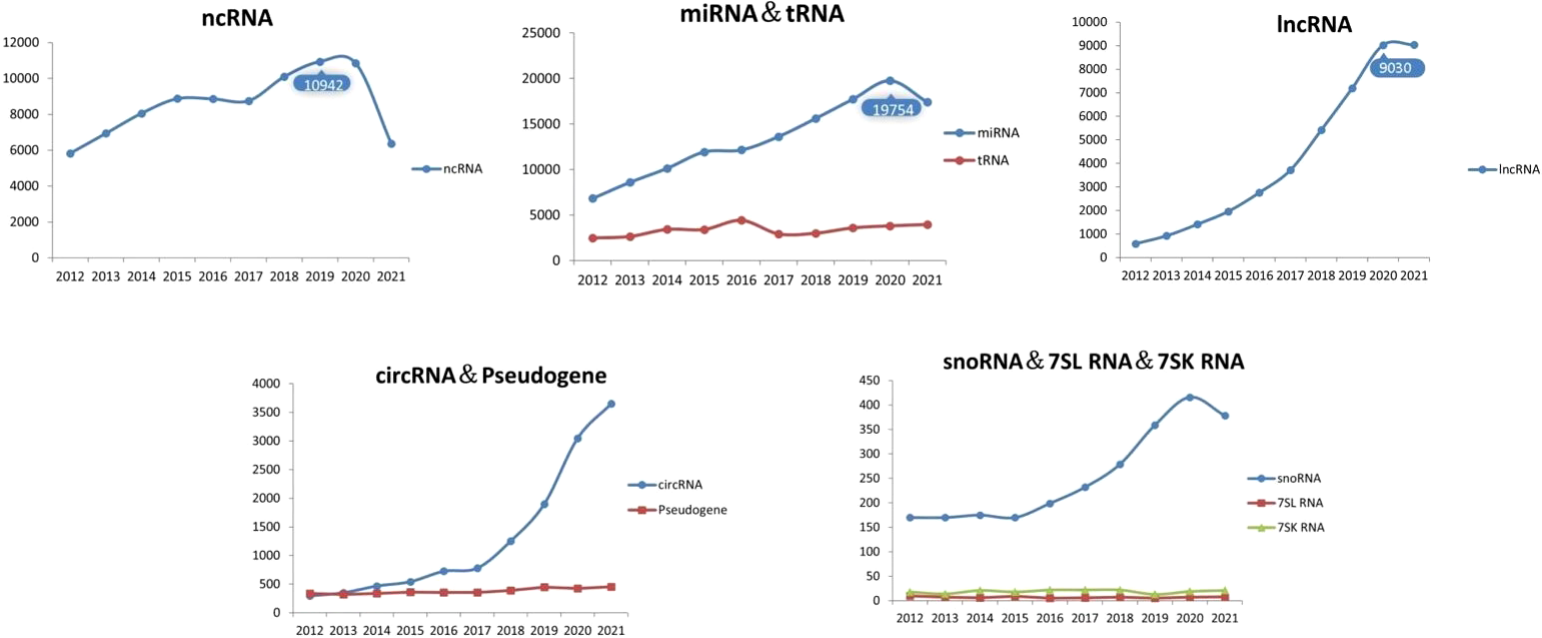
Number of articles related to ncRNAs.

Exosomes are tiny vesicles secreted by functionally normal or abnormal cells in the form of outgrowths and are a means of communication between cells. There are many substances included in the exosomes, such as the DNA, miRNA, lncRNA and proteins (32). Exosomes have a lipid bilayer structure and their production is regulated by multiple mechanisms involving the intra- and extracellular and microenvironment. Researchers have detected the presence of exosomes in various body fluids such as blood, saliva and urine. The characteristic structural proteins of exosomes, CD9, CD63, CD81, TSG101 and HSP70, are involved in the structural composition of exosomes. In addition, annexin, RAB protein family and flocculins are involved in biosynthesis and fusion (33, 34). The production process of exosomes is: the cell membrane is invaginated and the endosomes are formed then forming multivesicular bodies (MVB), and finally secreted to the outside of the cell to become exosomes (11). The structure and secretion mode of exosomes are shown in **Figure 1**. The contents of exosomes are different due to their different functions, and the origin of exosomes can be identified according to their contents (35). Exosomes are enriched with a variety of ncRNAs with biological functions, including lncRNAs, circRNAs, snRNAs, miRNAs, etc., which are important for the treatment of diseases. With the development of biology and other research fields, genomic ncRNAs are receiving more and more attention (36). A PubMed search shows a large number of scientific studies involving ncRNAs in the last decade, as shown in **Figure 2**. NcRNAs are RNA molecules that are transcribed from DNA, but not translated into proteins. NcRNAs used to be thought to be nonfunctional, but in recent years, functional ncRNAs have played important roles in higher order chromosome dynamics, embryonic stem cell differentiation, telomere biology, and subcellular structure. The potential properties of ncRNAs as a diagnostic and healing biomarker or therapeutic target for cancer have also been extensively studied (37).

Tumor cells secrete exosomal ncRNAs that form RNA-induced gene silencing complexes (RISCs), which effectively silence mRNAs at high rates in recipient cells and reprogram the transcriptome (38). Among the exosome-encapsulated ncRNAs, miRNAs is a universal non-coding single-stranded RNA that controls post-transcriptional gene expression by binding to complementary sequences in the 3 ‘untranslated region (UTR) of the target messenger RNA (mRNA) (39). Specific miRNAs expression and interference can be used to study the role of miRNAs in cancer development and progression (40). MiRNAs is closely related to cell development, proliferation, apoptosis, aging, carcinogenesis, lipid metabolism and viral infection (41). During tumor formation, overexpressed miRNAs can act as oncogenes, and conversely, low-expressed miRNAs are considered as tumor suppressor genes. It is worth mentioning that miRNAs are not randomly present among exosomes, but are directed to specific exosomes by a specific sorting mechanism dominated by parental cells. Therefore, there is a significant difference in exosomal miRNAs expression between normal and tumor tissues during cancer diagnosis, making miRNAs specific biomarkers designed for application in targeted cancer therapy (42). Exosomal lncRNAs can also serve as biomarkers of tumors to provide help for tumor therapy. Exosomal AFAP1-AS1 could induce trastuzumab resistance by binding to AUF1 and promoting ERBB2 translation. Therefore the level of AFAP1-AS1 may be used to predict trastuzumab resistance and breast cancer treatment (43). Consequently, the study of ncRNAs in tumor cell exosomes is of great significance for the diagnosis and treatment of tumors.

## Natural drugs regulate tumor genesis and development by regulating exosomal ncRNAs

Tumor development is inseparable from the TME (44, 45). Altering the TME and inhibiting angiogenesis directly affects the migration and invasion of tumor cells (46, 47). In addition, tumor cells are often accompanied by abnormal mechanisms of apoptosis, and selective induction of apoptosis using modern research techniques is also a fundamental strategy for cancer treatment. Exosomal ncRNAs, as outstanding antitumor factors, have been well documented to interfere with cancer progression and many other aspects (48). **Table 1** lists detailed examples of natural products that regulate exosomal ncRNAs to influence cancer progression.

**Table 1:** Detailed example of natural products affecting tumor exosomal ncRNAs.

Natural Product	Types of cancer/Associated with cancer	ncRNA	Target Genes	Related Hallmark	Reference
Matrine	Colorectal Cancer	circSLC7A6	Matrine	Inhibits tumorigenesis	49
Emblica officinalis	Ovarian Cancer	miR-375	IGF1R/SNAIL1	Regulation of TME	50
Rapamycin	Liver Fibrosis	miR-223	TGF-β	Suppressing autophagy	51
Epigallocatechin gallate	Cancer	miR-16	NF-κB	Inti-TAM/Inti-M2 polarization	52
Sulforaphane	Ductal Carcinoma In Situ	miR-140	ALDH1	Reduce cell colonies	53
Docetaxel	Breast Cancer	miR-9-5p,miR-195-5p,miR-203a-3p	ONECUT2	Dormant cells	53
Resveratrol	Liver Cancer	lncRNA SNHG29	Wnt/β-catenin	Anti-autophagy	54
G-rg1	Cancer blood vessels	miR-126-5P, miR-146a-5P,miR-210, miR-214-5P	VEGF	Regulate endothelial angiogenesis	55
Docosahexaenoic acid	Lymphoma	miR-34a,miR-125b,miR-221,miR-222/miR-9,miR-17-5p,miR-19,miR-126,miR-130,miR-132,miR-296,miR-378	VEGF	Anti-angiogenesis	56
Astragaloside IV	Cancer	miRNA-126	Exosome	Improve secretory exosomes	57
D Rhamnose β-hederin	Breast Cancer	miR-130a,miR-425	Exosome	Anti-proliferation	58
Shikonin	Breast Cancer	miR-128	Bax	Anti-proliferation	59
Lipopolysaccharide	Colorectal Cancer	miR-200c-3p	ZEB-1	Anti-migration/invasion/Anti-proliferation	60
Propofol	Liver Cancer	lncRNA H19,miR-520a-3p	LIMK1	Promoted the proliferation/Migration and invasion/Inhibited the apoptosis	61
Tanshinone II A	Laryngocarcinoma	miR-656-3p	apoptin	Anti-proliferation and invasion	62
Olive oil polyphenol hydroxytyrosol	Chronic Inflammation	miR-155-5p,miR-34a-5p,let-7c-5p	NF-κB	Anti- recruitment	63
Tazemetostat	Lymphoma	miR-378a-3p,miR-378d	Dickkopf 3/NUMB	Regulatory resistance	64
Docetaxel	Tongue Squamous Cell Aarcinoma	miR-200c	TUBB3/PPP2R1B	Regulatory resistance/Migration and invasion	65
Taxane	Ovarian Cancer	miR-200c	TUBB3	Regulatory resistance	65
Docetaxel	Ovarian Cancer	miR-146a	LAMC2	Regulatory resistance	66b
Docetaxel	Prostate Cancer	circ-XIAP,miR-1182	TPD52	Regulatory resistance	67
Docetaxel	Prostate Cancer	miR-27a	P53	Regulatory resistance	27
D Rhamnose β-hederin	Breast Cancer	miR-16,miR-23a,miR-24, miR-26a,miR-27a	D/exo	Regulatory resistance	68
β-elemene	Breast Cancer	miR-34a,miR-452	PTEN/Pgp	Regulatory resistance	69
Adriamycin	Breast Cancer	H19	miR-152/DNMT1	Decrease cell viability/Induction of apoptosis/Regulatory resistance	70

### Natural drugs improve the TME by regulating exosomal ncRNAs

**Figure 3:**
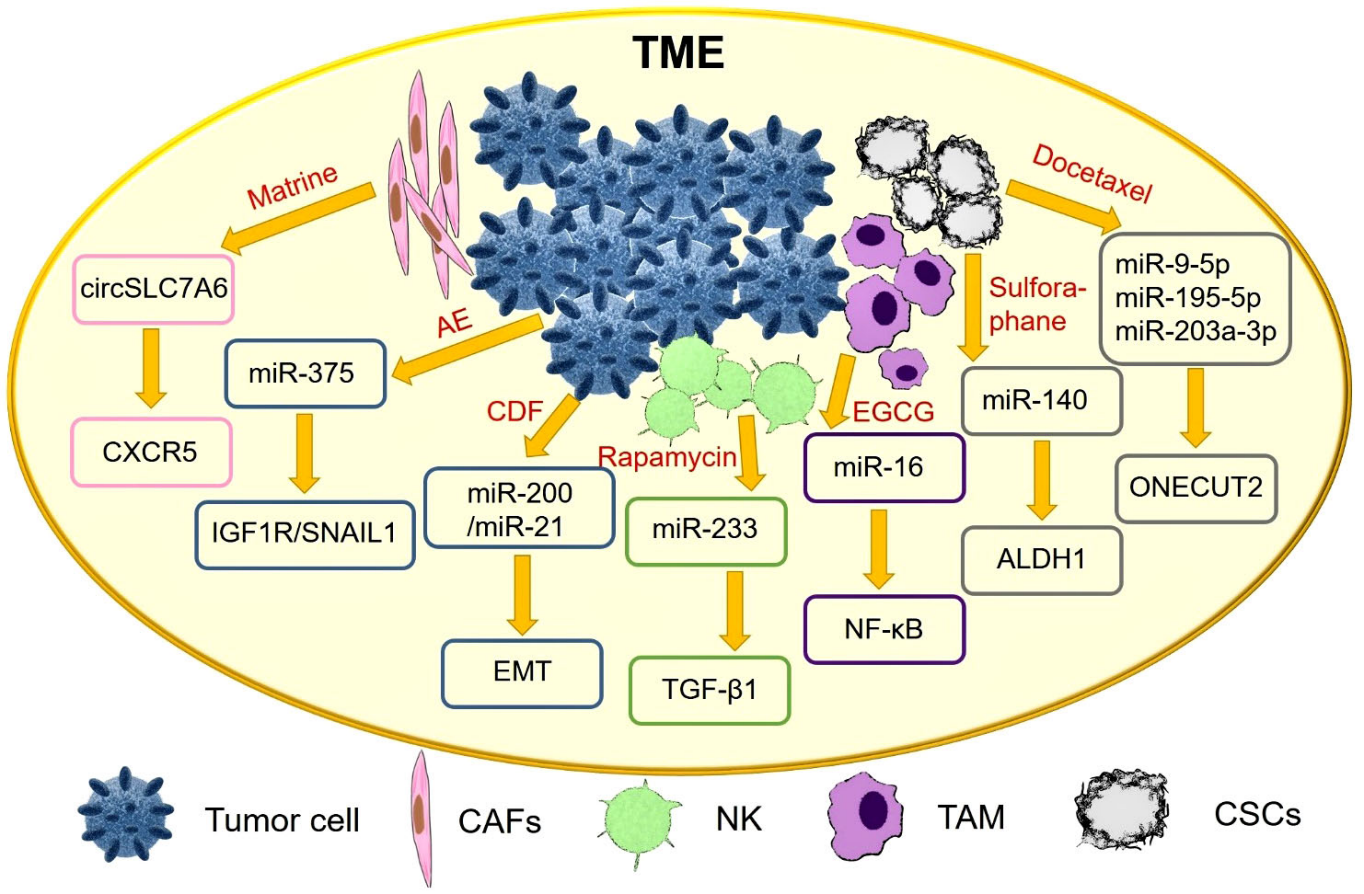
Natural drugs improve the TME by regulating exosomal ncRNAs.

Tumor cells and their microenvironment are a functional whole and both interact with each other to promote tumorigenesis (71). TME plays an important role in tumor growth, metabolism and metastasis, and even influences the therapeutic effect of anti-cancer drugs (72). In recent years, an increasing number of natural products exert anti-cancer effects by interfering with the TME, as shown in detail in **Figure 3**.

Cancer-associated fibroblasts (CAFs) are considered to be one of the key stromal cells of tumors. It has been reported that Matrine inhibit CRC tumorigenesis by blocking the release of CAFs exosome circSLC7A6, and chemokine receptor CXCR5 is a key effector of circSLC7A6 in regulating tumor formation (49).

*In vitro* experiments showed that Indian currant extract (Emblica officinalis, AE) upregulated miR-375 and adhesion protein e-cadherin, and downregulated insulin-like growth factor 1 receptor (IGF1R) and epithelial mesenchymal transition (EMT) factor SNAIL1. AE targets IGF1R and SNAIL1 by activating miR-375 in osteoblasts (50).

Activation of hepatic stellate cells (HSC) is an important driver of liver fibrosis and is closely associated with the formation of hepatocellular carcinoma. Exosomes from natural killer (NK) cells attenuate TGF-β 1-induced stellate cell activation. The secondary metabolite rapamycin, secreted by Streptomyces soil, inhibits miR-223 expression in natural killer exosomes (NK-EXO) (51).

Tumor-associated macrophages (TAM) play an important role in the TME. In particular, NF-κB expression affects M2 macrophages and promotes tumor progression. Tumor-derived exosomes can regulate the TME by transferring miRNAs to immune cells. Gallate (EGCG) upregulates exosomal miR-16 secreted by tumor cells, prevents its metastasis to TAM, and inhibits TAM infiltration and M2 polarization (52).

Basal-like ductal carcinoma *in situ* (DCIS) contains cancer stem cell-like cells (CSCs) with a high migration potential. Sulforaphane targets DCIS stem cell-like cells, decreases aldehyde dehydrogenase 1 (ALDH1) expression, and reduces mammary and progenitor cell colony formation. Differentially expressed miR-140 in exosomes secreted by DCIS affects signal transduction in nearby breast cancer cells (53).

Docetaxel increased the expression of miR-9-5p, miR-195-5p, and miR-203A-3p in circulating extracellular vesicles (EVs), decreased ONECUT2 expression, and increased the levels of related genes in xenograft mammary tumor-bearing mice (53). It has also been reported that resveratrol-mediated exosomal lncRNAs SNHG29 inhibit HCC progression by suppressing autophagy and Wnt/β-catenin pathway activation (54).

### Natural drugs regulate exosomal ncRNAs to inhibit angiogenesis

Angiogenesis is critical to the development of cancer and is the primary means of nutrient acquisition for tumors. Without vascular supply, tumors will not exceed 2 mm in diameter, which is the result of poor metabolism caused by inadequate nutrient and oxygen supply (46). Research has demonstrated that inhibition of angiogenesis can inhibit tumor growth and metastasis (73). Thus angiogenesis has emerged as a target for cancer therapy (74).

Xiong et al. proposed the effects of ginsenoside RG-1 and astragaloside on angiogenesis. Among them, ginsenoside RG-1 has the property of mediating miRNA-126-5P, miRNA-146A-5P, miRNA-210 and miRNA-214-5P to regulate endothelial angiogenesis (55), while Astragaloside IV increased human endothelial progenitor cells (EPC) secretion of exosomal miRNA-126-3p and miRNA-126-5p expression and promote angiogenesis (57).

Docosahexaenoic acid (DHA, ω 3:22-6) is a known omega-3 fatty acid derived from animal (e.g., fish oil) and plant sources (e.g., flaxseed oil) that plays an important role in influencing angiogenesis. DHA treatment of cells increased the expression levels of tumor suppressors’ miR-101, miR-199 and miR-342 and decreased the expression levels of miR-382 and miR-21. The secretion of exosomes was significantly reduced by DHA treatment under either normal or hypoxic conditions (75). The exosomal let-7a, miR -23b, miR -27a/b, miR -21, let-7 and miR -320b have anti-cancer or anti-angiogenic activity. DHA inhibits angiogenesis by altering exosome secretion and miRNA content in breast cancer cell lines (MDA-MB-231, ZR751 and BT20). After DHA treatment of MCF7 cells, their exosomes were applied directly to endothelial cell culture, and the expression of exosomal miRNAs in endothelial cells was increased (76). DHA treatment has been reported to lead to a significant reduction in VEGF expression and secretion in BC cells. The expression of anti-angiogenic miRNAs (miR-34a, miR-125b, miR-221 and miR-222) was increased and the expression of pro-angiogenic miRNAs (i.e. miR-9, miR-17-5p, miR-19a, miR-126, miR-130a, miR -132, miR-296 and miR-378) was decreased in TDE (DHA+) exosomes of DHA-treated BC cells. DHA reverses the therapeutic efficacy of these miRNAs from promoting angiogenesis to inhibiting angiogenesis by upregulating the content of exosomal miRNAs to achieve the purpose of modifying angiogenesis (56). All the above studies indicate that natural drugs have more complex forms of action, and people need to conduct deeper research on their pharmacological effects in order to achieve targeted treatment of diseases.

### Natural drugs regulate exosomes ncRNA to promote tumor cell apoptosis or inhibit proliferation

Inhibition of apoptosis is an important basis for tumorigenesis, which disrupts the balance between cell proliferation and apoptosis in normal tissues (77). Since the body cannot normally carry out the process of cell proliferation and apoptosis, the number of cells continues to increase, resulting in the formation of tumors. For tumors with the same proliferative capacity, the decrease in apoptosis rate also increases the net growth rate of tumor cells (78).

**Figure 4:**
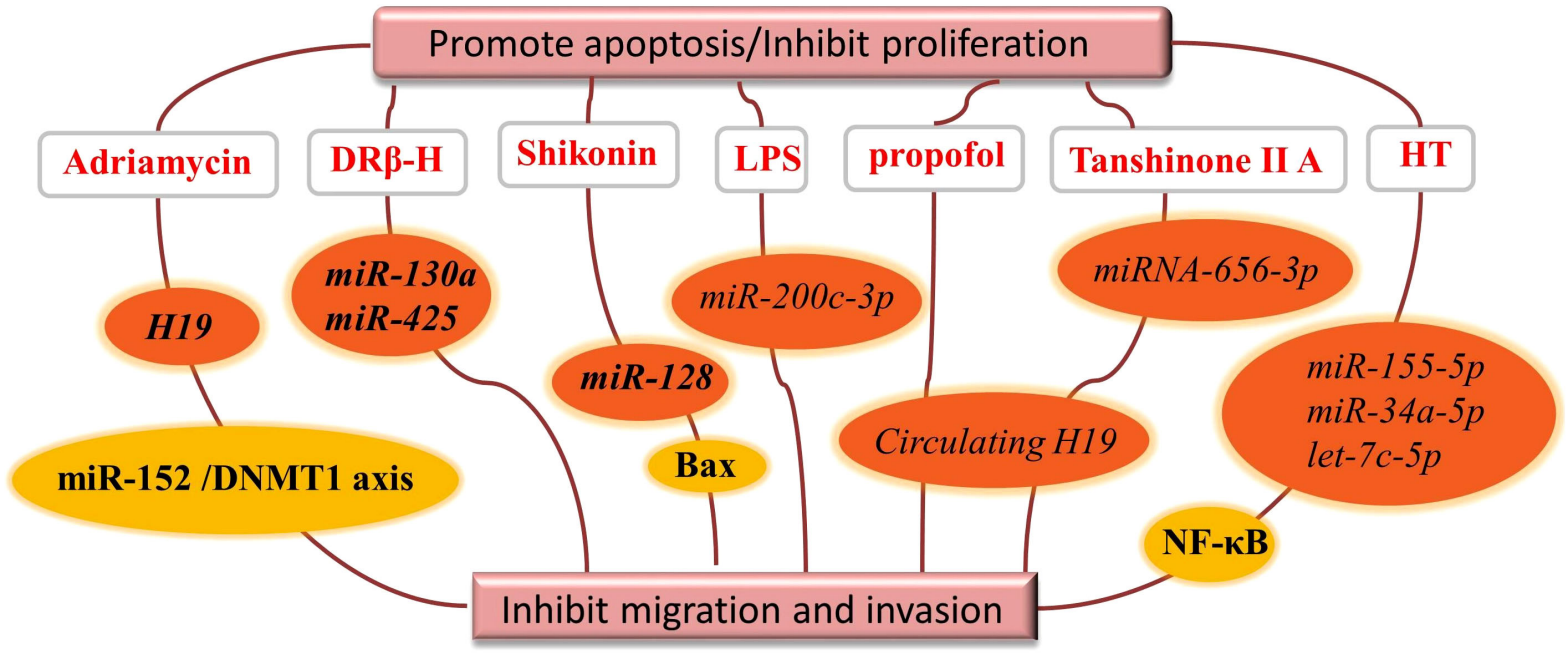
Natural drugs regulate exosomal ncRNAs to promote tumor cell apoptosis or inhibit.

Natural products inhibit tumor cell proliferation by regulating exosomal ncRNAs as shown in detail in **Figure 4**. The active ingredient d Rhamnose β-hederin (DRβ-H) showed anti-proliferative and pro-apoptotic activities in human breast cancer cells (McF-7/S), and DRβ-H inhibited the growth of human breast cancer cells by suppressing the secretion of exosomal miR-130a and miR-425 (58).

Shikonin is a naphthoquinone isolated from the traditional Chinese medicine Lithospermum, inhibits the proliferation of McF-7 breast cancer cells by reducing the tumor-derived exosomal miR-128, which has the property of negatively regulating Bax levels in McF-7 receptor cells (59).

Lipopolysaccharide (LPS) is an abundant component of the gut microbiota and has now been shown to be involved in CRC progression and metastasis by regulating exosomal miRNAs composition of CRC origin. In the presence of LPS, exosomal miR-200c-3p negatively regulates the migration and invasive ability of HCT-116 cells and promotes their apoptosis (52).

During the development of hepatocellular carcinoma, circulating H19 promotes proliferation, migration and invasion and inhibits apoptosis in isoproterenol-treated hepatocellular carcinoma cells by sponging miR-520a-3p upregulating LIMK1 (61). Tanshinone IIA also upregulated the expression level of miR-656-3p in the exosomes of CAFs and inhibited the proliferation and invasion of laryngeal cancer cells (62).

We know that chronic inflammation can induce a range of inflammatory diseases, including malignancies (79). A review of the literature revealed that natural products affect the inflammatory response by regulating exosomal ncRNAs. For example, olive oil polyphenol hydroxytyrosol (HT) significantly abolished the expression of miR-155-5p, miR-34a-5p and let-7c-5p in cells and exosomes and inhibited NF-κB activation and reactive oxygen species production. HT inhibited macrophage aggregation and improved chronic inflammatory inflammation in adipose tissue. MiR-155 was found to be an important factor in hepatocyte and macrophage in autophagy and exosome production of alcohol-related mediators (63).

Nowadays, the extensively studied natural fluorescent anthracycline antibiotic Adriamycin is a broad-spectrum antitumor antibiotic produced by the actinomycete Caesius. Increased expression of long-stranded ncRNA H19 in adriamycin-resistant breast cancer cells compared to the corresponding parental cells reduced cell viability and colony formation capacity and induced apoptosis. Another study pointed out that exosomal H19 promotes proliferation and invasion of breast cancer using miR-152/DNMT1 axis, providing a new mechanism for breast cancer development (70, 80).

Altogether, extensive evidence suggests that the pro-apoptotic and anti-proliferative properties of natural drugs are at least partially derived from exosomal ncRNAs.

### Natural drugs regulate exosomal ncRNAs to regulate drug resistance in tumor cells

Compared to normal patients, cancer patients secrete different levels of ncRNAs, which are ideal biomarkers for predicting early disease progression and drug resistance in cancer (81). Researchers have found that many ncRNAs expression profiles associated with drug resistance in tumor cells are concentrated in exosomes, and that exosomal ncRNAs can alter chemosensitivity, in part due to the successful transfer of multidrug resistance (MDR)-specific miRNAs between cells (82).

Docetaxel (DTX) is a semisynthetic product of precursors extracted from the needles of redbud (t. b accata L. Taxus). It has been reported that breast cancer cells resistant to DTX can pass on drug resistance by transferring specific miRNAs contained in exosomes to alter gene expression in sensitive cells (83). Exosomal miR-200c secreted by normal tongue epithelial cells (NTECs) is transported to HSC-3DR, making HSC-3DR more sensitive to DTX by binding TUBB3 and PPP2R1B. MiR-200c regulates HSC-3DR sensitivity to DTX by targeting TUBB3 and PPP2R1B. The researchers concluded that exosome-mediated miR-200c delivery may be an effective and promising strategy for the treatment of tongue squamous cell chemoresistance (84). In ovarian cancer, miR-200c sensitizes tumor cells to paclitaxel by binding TUBB3. Overexpression of miR-200c reversed cell resistance to DTX-mediated migration and invasion (65).In addition, exosomal miRNA-146a from mesenchymal stem cells (MSCs) increased the sensitivity of ovarian cancer cells to DTX and paclitaxel *via* the lamcc2-mediated PI3K/Akt axis (66). Exosomal CIRC-XIAP promotes DTX resistance of PCa by regulating miR-1182/TPD52 axis, providing a promising therapeutic target for PCa chemotherapy (67). It was also reported that treatment of SCS -27 cells with DTX resulted in a significant increase in the level of exosomal miR-27a expression and inhibition of P53 gene expression, which contributed to the enhancement of chemoresistance in PCa (27).Several abundant miRNAs (miR-16, miR-23a, miR-24, miR-26a and miR-27a) were transported by exosomes secreted by breast cancer cells McF-7. DRβ-H extracted from the traditional Chinese medicinal plant Clematis ganpiniana, was able to reduce the expression of miR-16, miR-23a, miR-24, miR-26a and miR-27a transported by D/exo and reverse the DTX resistance (68).

New studies have shown that exosomal ncRNAs are mediators of intercellular communication between heterogeneous tumor cell populations, and that some exosomal ncRNAs promote tumor cell resistance to natural products during the communication process, and that only inhibition of constant shuttling of ncRNAs can improve tumor cell drug resistance (85). In the treatment of breast cancer, β-elemene mediates MDR-related miR-34a and miR-452 in cells and regulates the expression of target genes PTEN and PGP, reducing chemoresistance transmission through exosomes and reversing drug resistance in breast cancer cells (86). Zeste homolog 2 (EZH2)-enhanced STAT3 binds to the promoter regions of miR-378a-3p and miR-378d, thereby increasing their expression in exosomes. Exosomes produced by BC cells after stimulation with DOX or PTX deliver miR-378a-3p and miR-378d to neighboring cells to activate the WNT and NOTCH stemness pathways and induce drug resistance by targeting Dickkopf 3 (DKK3) and NUMB. Tazemetostat, an EZH2 inhibitor, is an epigenetic drug whose combination with chemotherapeutic agents can reverse chemotherapy-induced resistance (64). Additionally, exosomal SNHG7 activated the phosphatidylinositol 3-kinase (PI3K)/AKT pathway to promote M2 polarization in macrophages *via* recruiting cullin 4A (CUL4A) to induce ubiquitination and degradation of phosphatase and tensin homolog (PTEN). Silencing of SNHG7 enhances the potency of doxorubicin and inhibits proliferation and autophagy in LUAD cells (84). Perhaps exosome-mediated resistance reversal is one of the mechanisms by which it acts as a vehicle to enhance the efficacy of drugs.

### Exosomes of natural origin for cancer prevention and treatment

Exosomes can act as natural products to exert pharmacological activity, that is, to target lesions. In existing studies, there are numerous reports of exosomes carrying ncRNAs that regulate relevant metabolic pathways to influence disease.

Exosomal miR-133b may inhibit tumor growth *in vivo* through upregulation of dual protein phosphatase 1 (DUSP1). Increased expression of exosomal miR-133b leads to inhibition of bladder cancer cell viability and increased apoptosis (87). MiR-139-5p is a mesenchymal stem cell-derived exosomal ncRNA that inhibits bladder cancer development *in vitro* and *in vivo* (88).Exosomal miR-499 not only significantly inhibited endometrial cancer cell proliferation and endothelial cell tube formation *in vitro*, but also tumor growth and angiogenesis *in vivo* (89). In addition, in exosomes derived from tumor-associated macrophages, miR-192-5p overexpression effectively inhibited EC progression by regulating EC apoptosis and EMT and inhibiting the IRAK1/NF-κB signaling pathway (90). CAF-secreted exosomal miR-320a was directly transferred to EC cells, inhibiting their proliferation and leading to downregulation of HIF1α and decreased VEGFA expression *in vitro* (91). For breast, lung, and oral squamous cell carcinomas, the normal cell-secreted exosomal PTENP1 mediates intercellular communication by promoting apoptosis in breast cancer cells as well as inhibiting invasion and migration (92).

Exosome-mediated intercellular communication influences several features of cancer, including regulation of the immune response, reprogramming of stromal cells, remodeling of the structure of the extracellular matrix, and even conferring characteristics of drug resistance to cancer cells (93, 94). Uptake of exophytic vesicles by tumor cells can alter gene expression and reduce cancer-associated phenotypes (95). Similar to animal exosomes, plant exosomal vesicles contain specific lipids, proteins, nucleic acids, and other components, each of which may play a role in the development of disease by performing the corresponding biological functions through specific mechanisms. Similar exosomal nanoparticles isolated from citrus limoncello juice inhibit lipid metabolism, leading to a significant downregulation of acetyl coenzyme a carboxylase 1 (ACACA), thereby inhibiting tumor cell growth *in vitro* and *in vivo* (96). Mango Nano Capsules inhibit chronic myeloid leukemia (CML) tumor growth *in vivo* by specifically reaching the tumor site and activating TRAIL-mediated apoptotic cell processes (97). Berry-derived anthocyanins significantly enhance their proliferative activity against ovarian cancer cell growth and inhibit tumor growth more effectively (98). Natural plant exosomes are rich in miRNAs, and research on miRNAs in common plants is expanding and intensifying. A growing number of studies suggest that exosomal miRNAs may interact with mammalian cancer-related systems as novel bioactive components. Ginger-derived nanoparticles are a novel and effective drug that blocks the assembly and activation of pyrane-structured domain NLRP3 inflammatory vesicles (99). Exosomal MDO-miR7267-3p ameliorates colitis in mice through an il-22-dependent mechanism (100), and regulate AhR expression by inducing miR-375 and VAMP7 to prevent high-fat diet-induced insulin resistance and obesity (101). In addition, ginger-derived nanoparticles also inhibited LPS-induced inflammatory responses through downregulation of NF-κβ, IL-6, IL-8 and TNF-α (102). In conclusion, numerous natural exosomes have been found to be used for cancer prevention and treatment, which provides new ideas for the clinical treatment of cancer.

## Exosomes ncRNAs as predictive biomarkers

Exosomes have potential as predictive biomarkers and play an important role in intercellular communication in cancer cells. MiRNAs inhibits translation of oncogenes and is involved in cancer development by regulating cell proliferation and differentiation (103). In some cases, dysregulated exosomal miRNAs can be used as “tumor markers” for disease diagnosis (103, 104). **Table 2** shows the expression of exosomal ncRNAs in high-incidence cancers.

**Table 2:** Detailed information on exosomal ncRNAs as biomarkers.

Types of cancer/Associated with cancer	ncRNA	Expression	Potential clinical value	Type of biomarker	Reference
DNA damage	lincRNA-p21, HOTAIR, ncRNA-CCND1	increased	Biomarker	Diagnosis	105
large B-cell lymphoma	miR-99a-5p, miR-125b-5p	increased	Biomarker	Diagnosis	106
large B-cell lymphoma	miR-483-3p, miR-451a	decreased	Biomarker	Diagnosis	107
large B-cell lymphoma	miR-379-5p, miR-135a-3p, miR-4476	increased	Biomarker	Diagnosis	107
Anaplastic large cell lymphoma Burkitt lymphoma Hodgkin lymphoma mature B-cell acute lymphoblastic leukemia	miR-191-5p	increased	Biomarker	Diagnosis	108
esophagus cancer	miR-21	increased	Biomarker	Diagnosis	69
glioblastoma	miR-21	increased	Biomarker	Diagnosis	109
colorectal cancer	let-7a, miR-1229, miR-1246, miR-150 miR-21, miR-223, miR-23a	increased	Biomarker	Diagnosis	110
Metastatic colorectal cancer	miR-25-3p	increased	Biomarker	Diagnosis	111
prostatic cancer	miR-1290, miR-375	increased	Biomarker	Prognosis	112
prostatic cancer	miR-99a-5p	decreased	Biomarker	Diagnosis	113
prostatic cancer	miR-21-5p	increased	Biomarker	Diagnosis	114
prostatic cancer	miR-200c-3p	decreased	Biomarker	Diagnosis	114
prostatic cancer	miR-96-5p, miR-183-5p	increased	Biomarker	Diagnosis	115
endometrial cancer	miR-15a-5p	increased	Biomarker	Diagnosis	116a
ovarian cancer	miR-21, miR141	increased	Biomarker	Diagnosis	117
melanoma	miR-494	increased	Biomarker	Diagnosis	118
melanoma	miR-1180-3p	decreased	Biomarker	Diagnosis	119
melanoma	miR-143, 221	increased	Biomarker	Diagnosis	120
DM-ILD-MDA5 Ab(+)	hsa-miR-1228-5p	increased	Biomarker	Diagnosis	121
DM-ILD-MDA6 Ab(+)	hsa-miR-4488	increased	Biomarker	Diagnosis	
renal cell carcinoma	miR-30c-5p	increased	Biomarker	Diagnosis	122
oral squamous cell carcinoma	miR-365	increased	Biomarker	Diagnosis	123
gastric carcinoma	miR-let-7	increased	Biomarker	Diagnosis	124
gastric carcinoma	miR-328-3p, miR-339-5p	increased	Biomarker	Prognosis	125
gastric carcinoma	miR-1-3p, miR-151a-3p, miR-184 miR-202-5p, miR-34c-5p, miR-3470a miR-3470b, miR-466i-5p	decreased	Biomarker	Prognosis	125
non-small cell lung cancer	let-7f, miR-20b, miR-30e-3p	decreased	Biomarker	Diagnosis	126
non-small cell lung cancer	hsa-miR-320d, hsa-miR-320c hsa-miR-320b	increased	Biomarker	Diagnosis	127
non-small cell lung cancer	hsa-miR-125b-5p	increased	Biomarker	Diagnosis	127
liver cancer	miRNA-21, lncRNA-ATB	increased	Biomarker	Prognosis	128
liver cancer	miR-34a	decreased	Therapeutic target	Prognosis	129

The expression of miRNAs in exosomes changed with altered physiological conditions. For example, when DNA damage was induced by the application of bleomycin, RNA molecules with relatively low expression levels (lincRNAs-p21, HOTAIR, ncRNAs-CCND1) were highly enriched in exosomes, reflecting changes in the expression levels of ncRNAs after cells are exposed to the drug (105). For diffuse large B-cell lymphoma, exosomal miR-99a-5p and miR-125b-5p can be used as predictive biomarkers of chemotherapy resistance (106). In contrast, the exosomal miR-483-3p and miR-451a serve as potential biomarkers for monitoring patient response to treatment (107). In lymphoma, exosomal miR-191-5p expression was higher (108). For colorectal cancer, a group of exosomal miRNAs including let-7a, miR-1229, miR-1246, miR-150, miR-21, miR-223 and miR-23a can be used as diagnostic biomarkers for colorectal cancer (110). And the expression level of exosomal miR-25-3p was also significantly correlated with colorectal cancer cell metastasis (111). Exosomal miR-21 is a potential marker for patients with esophageal cancer (69). Similarly, its level in glioblastoma is higher than normal (109). Urinary miR-21-5p and miR-200c-3p can be used as potential non-invasive biomarkers for prostate cancer patients (114). Another group of miR-1290 and miR-375 could be used as prognostic markers for trend resistant prostate cancer (112). Meanwhile, miR-99a-5p was downregulated in prostate tumor tissues (113). In breast cancer patients, urinary exosomal miR-96-5p and miR-183-5p expression levels were higher (115). Plasma-derived exosomal miR-15a-5p has been reported to be a promising biomarker for the diagnosis of endometrial cancer (116), and the expression levels of miR-21 and miR141 in benign tumor exosomes were different from those in ovarian cancer (117). For melanoma cells, increased expression of exosomal miR-494 and downregulated expression of Rab27A inhibited tumor growth and metastasis (118). Besides, the level of exosomal miR-1180-3p was negatively correlated with the proliferation, migration and invasion of melanoma cells, and reduced the high expression of ST3GAL4 in melanoma cells (119). In addition, miR-143 and miR-221 were significantly increased in plasma exosomes of patients with metastatic melanoma (120). In patients with dermatomyositis (DM)-associated interstitial lung disease (ILD) combined with anti-melanoma differentiation-associated protein 5 antibody (MDA5), compared with myositis-specific antibody-negative patients without ILD (DM-nonILD - msa16(-)) and normal human controls (HC), hsa- miR-4488 was significantly upregulated and hsa-miR-1228-5P was upregulated in DM-ILD-MDA5 Ab(+) and downregulated in DM-nonILD-MSA16(-) compared to HC (121). Song et al. reported that urinary exosomal miR-30C-5p overexpression inhibited the progression of renal cell carcinoma (ccRCC). MiR-30c-5p prevented the depletion of heat shock protein 5 and reversed the growth-promoting effect of ccRCC (122). Elevated miR-365 levels in oral squamous cell carcinoma exosomes can be used as a potential biomarker for oral squamous cell carcinoma exosomes and EVs (123). In studies related to gastric cancer, members of the exosomal miR-let-7 family were most abundantly expressed in AZ-P7A cells (124). Others explored the changes in the expression of exosomes miR-328-3p, miR-339-5p, miR-1-3p, miR-151a-3p, miR-184, miR-202-5p, miR-34c-5p, miR-3470a, miR-3470b, and miR-466i-5p in gastric cancer cells (125). Hsa-miR-320d, hsa-miR-320c and hsa-miR-320b are considered potential biomarkers for predicting the efficacy of immunotherapy in advanced NSCLC, and the levels of let-7f, miR-20b and miR-30e-3p are lower in plasma vesicles of NSCLC patients than in normal controls (126), when the T-cell suppressor hsa-miR-125b-5p was downregulated during treatment, patients had increased T-cell function and responded well to immunotherapy (127). Circulating exosomal miRNA-21 and lncRNAs-ATB are new prognostic markers and therapeutic targets for HCC (128). In addition, exosomal circRNAs secreted by adipocytes promoted HCC tumor growth and reduced DNA damage by inhibiting miR-34a and activating the USP7/Cyclin A2 signaling pathway (129).

Exosomes carry a wealth of bioinformatics molecules and are actively involved in mediating a variety of biological functions. A growing number of studies have shown that the use of exosomes as therapeutic targets for cancer can improve the efficiency of cancer treatment. For example, metastasis of exosomal MDR-1/P-GP excreted by prostate cancer cells resulted in docetaxel resistance to DU145, 22Rv1 and LNCap cells. Serum exosomes induced increased cell proliferation and invasion in prostate cancer patients compared to controls (130). Signaling between preadipocytes and breast cancer cells has been found to promote breast tumor formation and metastasis. Exosomes secreted by preadipocytes are an important component of the tumor stem cell ecotone and regulate differentiation and migration in the TME through the critical miR-140/SOX2/SOX9 axis. Targeting exosome-related signaling may help to block tumor progression (131). And cd147-positive exosomes from epithelial ovarian cancer cells promote endothelial angiogenesis *in vitro* (132). In summary, we speculate that exosomal ncRNAs will develop in two directions in the future: firstly, as a new clinical malignancy marker to help timely detection and diagnosis of cancer, and secondly, as a tumor therapeutic target to help alleviate clinical disease symptoms.

## Discussion

Natural products have biomolecules and drug targets that have natural affinities and natural possibilities to participate in various physiological processes in living organisms, which will contribute to the discovery of additional and deeper drug mechanisms.

For tumor treatment, the development of new drug delivery strategies to accurately eliminate cancer cells has become the focus of conquering tumors. At present, the carriers of natural drugs such as curcumin and vogonin in the treatment of cancer include liposomes (133, 134), phospholipid complexes, emulsion systems (135) and metastases (136, 137). However, these drug delivery systems have problems such as low utilization rate and incomplete efficacy. Exosomes have the advantages of low immunogenicity, good biocompatibility, high drug-carrying capacity and long life span, and tumor cell-derived exosomes have great potential as drug carriers for targeting parental tumors (138–143). One investigator used DTX to select effective exosome loads by electroporation to deliver small molecules and siRNAs to tumor sites. (144).

In addition to acting as a vector for drugs to increase efficacy, exosomes themselves can be involved in the diagnosis and treatment of cancer as markers or anti-cancer drugs. In the TME, exosomes selectively wrap ncRNAs and are involved in cell-cell information exchange. Exosomes promote cancer progression and migration through this cell-to-cell communication. For instance, exosome MicroRNA-103 can promote proliferation and invasion of liver cancer cells (145). Numerous studies have shown that abnormalities in the secretion of exosomes occur during the development of cancer, providing a theoretical basis for the use of exosomes in early cancer screening (146). Recent studies have found that exosomes derived from bone marrow MSCs carry ncRNAs that can suppress cancer, including lncRNA PTENP1, which can suppress bladder cancer, and microRNA-551b-3p, which can suppress breast cancer. (147, 148).

Natural products as drugs have advantages such as low toxicity and multiple targets. Drugs found in natural products such as paclitaxel and pergolide have excellent therapeutic effects, but their mechanisms of action are not fully understood, and many natural products and traditional herbs are believed to have good anti-tumor effects. The vast majority of studies have discussed the role of natural products in inhibiting cell migration and value addition during tumorigenesis, and also discussed the role of natural products in tumor microenvironment and immune regulation, but their specific anti-cancer mechanisms have not been elucidated. Natural products are complex in composition and have multiple targets in cancer. Great progress has been made in recent years in the research of anti-tumor mechanisms of various monomeric components, such as curcumin, berberine, resveratrol, etc. The combination of these drugs with clinical anticancer drugs can delay the survival time of patients, reduce chemotherapy drug dosing, reverse drug resistance, and alleviate adverse effects. Relevant technologies regarding new drug development are constantly being improved, the level of drug research is increasing, and potential antitumor natural products are being explored. It is crucial to further elucidate the anticancer pharmacological effects of natural products and to give full play to their anticancer advantages. Exosomal ncRNAs are involved in the physiological and pathological processes of cancer by directly or indirectly regulating the expression of target genes, and play a key role in transcription and translation. The development of targeted anti-cancer drugs using exosomes not only can break the blood-brain barrier, but also avoid immune rejection by the human body (149).

Exosomes have a lipid bilayer structure that protects ncRNAs from degradation by ribonucleases, thus preserving their functional activity and facilitating specimen collection and storage. The enrichment of specific signaling molecules within the exosome membrane is more conducive to detection, as well as deep involvement in tumor events and closer to tumor nature, which is clinically important for achieving minimally invasive detection and early diagnosis and treatment of cancer. Compared to tumor exosome-specific proteins and lipids, exosomal ncRNAs have higher sensitivity and specificity and can be repeatedly sampled for large-scale assays.

Exosomal ncRNAs are a foothold for the development of new cancer diagnostic and therapeutic models, and it is important to identify and isolate exosomes secreted by tumor cells. However, at present, the technology of exosome isolation and purification is not mature enough to ensure the purity and activity of exosomes, and the establishment of a stable method for isolation and storage of exosomes is still an urgent problem. In addition, exosomes are secreted by a variety of cells, and it remains to be studied how to determine their source and the amount of ncRNA in exosomes is limited, and the technique to allow exosomes to precisely reach the tumor site needs to be improved. After literature survey, we found that the regulatory mechanisms of natural products using exosomal ncRNAs against cancer still need to be exhaustively demonstrated, and the specificity of exosomal ncRNAs as molecular markers for cancer diagnosis cannot be determined yet, and it is still difficult to fully resolve their regulatory networks. With the resolution of these unknown issues, the therapeutic application of exosomal ncRNAs in clinical practice for disease treatment will be realized soon.

## Author contributions

SH was responsible for the writing of the manuscript, YL for the editing of the graphs and charts, SG for the editing and recording of the tables, and ZQ and DL for the revision and final review of the manuscript. All authors were involved in the creation and were responsible for the content of the work.

## Funding

This work was supported by the National Natural Science Foundation of China (Grant No. 81973712, 82003985). Jilin Province Science and Technology Development Project in China (Grant No. 20210204013YY). Jilin Province Science and Technology Development Plan Project (Grant No. 20200708081YY).

## Conflict of interest

The authors declare that the research was conducted in the absence of any commercial or financial relationships that could be construed as a potential conflict of interest.

## Publisher’s note

All claims expressed in this article are solely those of the authors and do not necessarily represent those of their affiliated organizations, or those of the publisher, the editors and the reviewers. Any product that may be evaluated in this article, or claim that may be made by its manufacturer, is not guaranteed or endorsed by the publisher.

## Abbreviations

ncRNA, non-coding RNA; lncRNA, long non-coding RNA; miRNA, microRNA; siRNA, small interfering RNA; rRNA, ribosomal RNA; tRNA, transfer RNA; snRNA, small nuclear RNA; EMT, epithelial-mesenchymal transition; ceRNA, competing endogenous RNA; circRNA, circular RNA; ACS, American Cancer Society; UTR, untranslated region; RISCs, RNA-induced gene silencing complexes; TME, tumor microenvironment; CAFs, cancer-associated fibroblasts; IGF1R, insulin-like growth factor 1 receptor; EMT, epithelial mesenchymal transition; HSC, hepatic stellate cells; NK, natural killer; TAM, tumor-associated macrophages; DCIS, ductal carcinoma in situ; ALDH1, aldehyde dehydrogenase 1; EPC, endothelial progenitor cells; DHA, Docosahexaenoic acid; DRβ-H, d Rhamnose β-hederin; LPS, Lipopolysaccharide; MDR, multidrug resistance; EZH2, zeste homolog 2; DKK3, dickkopf 3; NTECs, normal tongue epithelial cells; EPC-EXS, Endothelial progenitor cell exosomes; DUSP1, dual protein phosphatase 1; ACACA, acetyl coenzyme a carboxylase 1; CML, chronic myeloid leukemia; ILD, interstitial lung disease; MDA5, melanoma differentiation-associated protein 5; ccRCC, renal cell carcinoma; TBI, traumatic brain injury; EVs, extracellular vesicles.
